# Inflammation and lipid-related determinants in coronary atherosclerosis: mechanisms, biomarkers, and therapeutic implications

**DOI:** 10.3389/fcvm.2026.1790509

**Published:** 2026-04-22

**Authors:** Fubo Zhang, Qingchi Liao

**Affiliations:** 1 The First School of Clinical Medicine, Faculty of Medicine, Yangzhou University, Yangzhou, China; 2 Northern Jiangsu People's Hospital Affiliated to Yangzhou University, Yangzhou, China

**Keywords:** apoB, coronary atherosclerosis, IL-6, inflammation, LDL-C, lipoprotein(a), NLRP3 inflammasome, remnant cholesterol

## Abstract

Coronary atherosclerosis is increasingly recognized as a chronic, maladaptive inflammatory disease initiated by arterial retention of apolipoprotein B (apoB)–containing lipoproteins and amplified by innate and adaptive immune responses. Although low-density lipoprotein cholesterol (LDL-C) remains a central causal factor, substantial residual risk persists despite intensive LDL-C lowering, emphasizing the clinical relevance of residual inflammatory risk and additional atherogenic lipid metrics such as apolipoprotein B (apoB), remnant cholesterol, small dense LDL, and lipoprotein(a) [Lp(a)]. Landmark outcome trials validate both paradigms: potent lipid-lowering therapies reduce major adverse cardiovascular events, and targeted anti-inflammatory therapies such as IL-1β inhibition and low-dose colchicine reduce recurrent events without altering LDL-C, establishing inflammation as a modifiable driver of coronary risk. This review integrates mechanistic evidence linking lipids and inflammation across the atherosclerotic continuum—from endothelial activation and leukocyte recruitment to plaque destabilization and thrombosis—while critically appraising biomarkers, imaging approaches, and therapeutic strategies. We propose a practical dual-axis framework integrating residual cholesterol and inflammatory risks to guide combined therapy and highlight future directions including genetics-informed lipid management [notably Lp(a)], inflammation-resolution biology, and precision targeting of upstream inflammatory pathways such as IL-6 signaling.

## 1 Introduction

**Figure 1:**
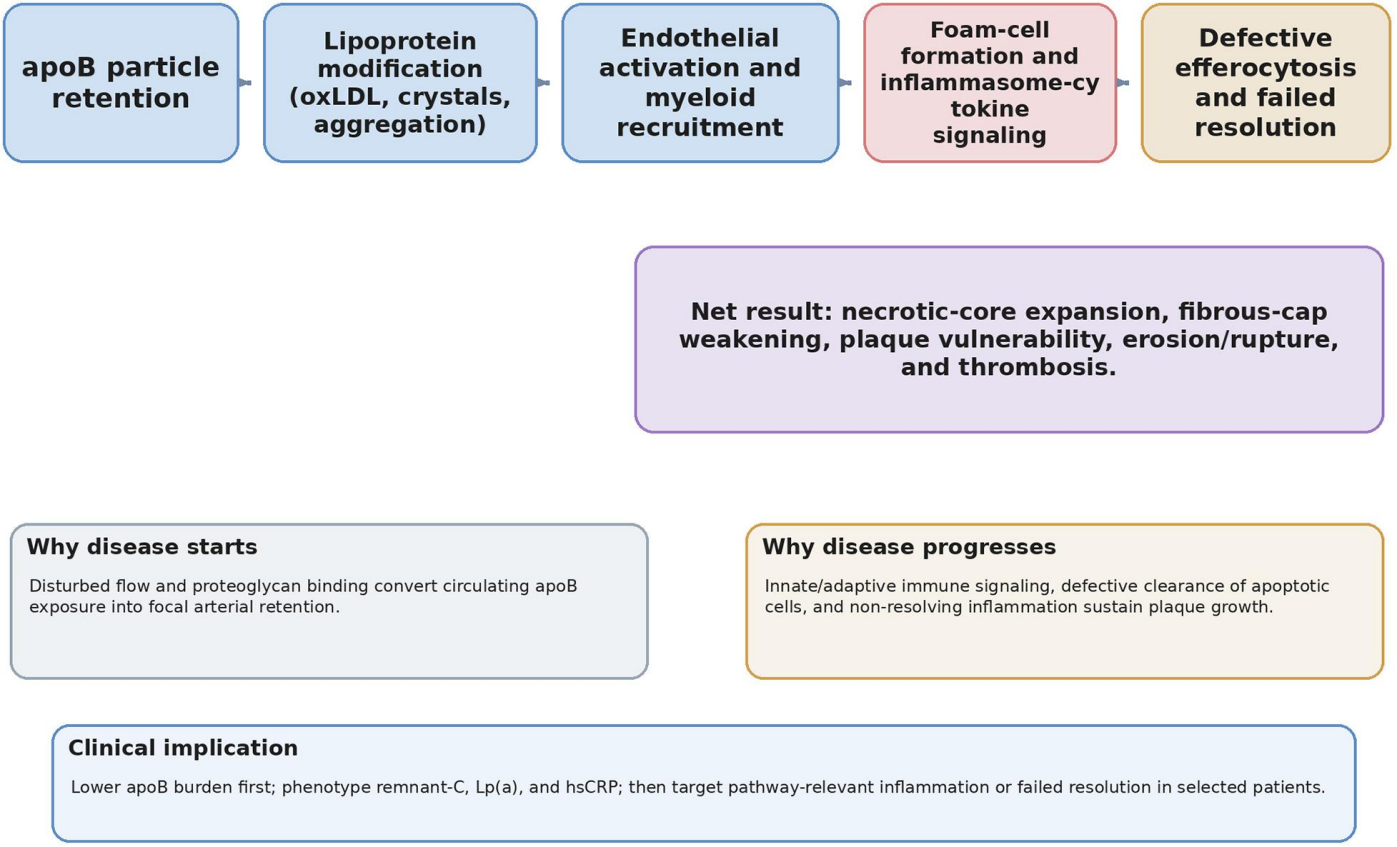
Integrated lipid–inflammation pathobiology in coronary atherosclerosis. ApoB particle retention and modification initiate endothelial activation, myeloid recruitment, foam-cell formation, inflammasome-cytokine signaling, failed resolution, and plaque vulnerability.

Coronary atherosclerosis is best understood as a chronic inflammatory disease initiated by arterial retention of apolipoprotein B (apoB)-containing lipoproteins and then amplified by innate and adaptive immune responses (1–16). Figure 1 summarizes this integrated lipid–inflammation continuum. Although low-density lipoprotein cholesterol (LDL-C) remains the dominant causal exposure and therapeutic target, substantial residual risk persists despite intensive LDL-C lowering, underscoring the need to interrogate apoB particle burden, remnant cholesterol, small dense LDL (sdLDL), lipoprotein(a) [Lp(a)], and persistent inflammatory activation when risk remains high.

**Figure 2:**
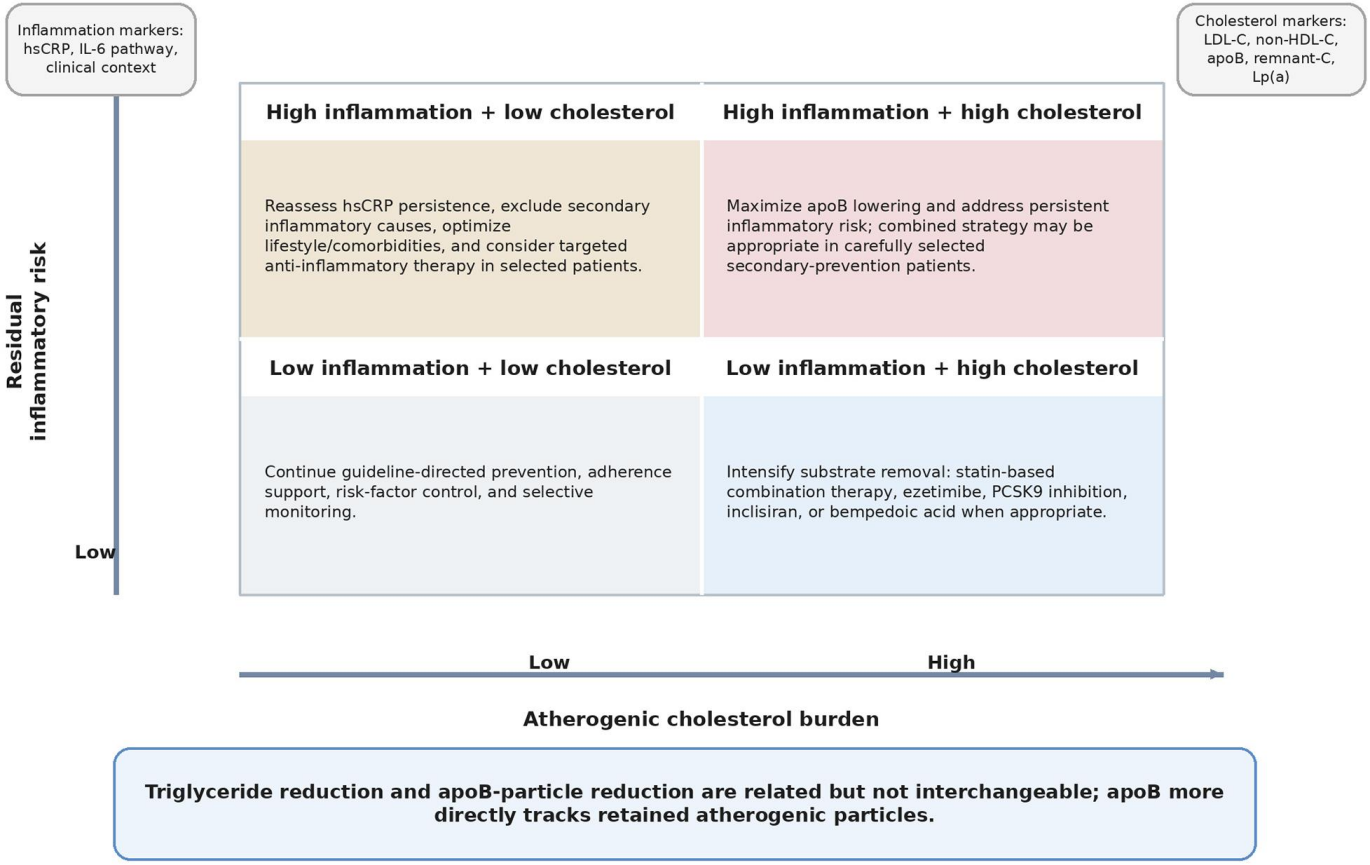
Dual-axis residual risk framework. Patients are stratified according to residual cholesterol risk and residual inflammatory risk to guide intensification of substrate lowering, targeted anti-inflammatory therapy, or combined treatment.

Within the response-to-retention model, subendothelial trapping of apoB-containing particles converts circulating lipoprotein exposure into local vascular injury and inflammation (11, 12). Figure 2 and Table 1 therefore frame this review around two clinically actionable axes: residual cholesterol risk and residual inflammatory risk. Retained particles undergo oxidation, aggregation, and enzymatic remodeling, which activate endothelial cells, recruit leukocytes, and reprogram macrophage immunometabolism; if inflammatory resolution and efferocytosis subsequently fail, necrotic core expansion, cap thinning, and plaque vulnerability follow.

**Table 1:** Lipid-related determinants, clinical metrics, and representative evidence.

Determinant	What it captures	Why it matters	Representative evidence
LDL-C	LDL cholesterol content	Causal driver; primary target	IMPROVE-IT; FOURIER; ODYSSEY OUTCOMES; CLEAR Outcomes
Non–HDL-C	Total atherogenic cholesterol	Captures LDL+remnants; robust with high TG	Guidelines; cohort evidence
ApoB	Atherogenic particle number	Reveals discordance; aligns with causal exposure	Discordance studies; statements
Remnant-C	TRL/remnant cholesterol content	Causal association; metabolic dyslipidemia risk	Genetics; PROMINENT context
sdLDL	Small dense LDL fraction	Oxidation-prone; metabolic marker	Meta-analyses
Lp(a)	Genetically determined apoB lipoprotein	Independent causal risk; therapies emerging	EAS consensus; RNA/oral therapies
HDL function	Cholesterol efflux capacity	Beyond HDL-C concentration	Prospective efflux studies

Landmark outcome trials validate both pillars of this paradigm. Potent lipid-lowering therapies (e.g., ezetimibe, PCSK9 inhibitors, bempedoic acid, and long-acting RNA-based approaches) reduce major adverse cardiovascular events (MACE) by lowering apoB exposure (7–10). Conversely, targeted anti-inflammatory strategies (e.g., IL-1β inhibition and low-dose colchicine) reduce recurrent events largely independent of LDL-C, establishing inflammation as a modifiable driver of coronary risk (11–14). Together, these data justify an integrated approach: treat residual cholesterol risk (particle burden and composition) and residual inflammatory risk (pathway-relevant inflammatory activity).

This review is organized to progress from causative biology to practical clinical use. Retained apoB-containing particles are treated as the initiating substrate, inflammation as the context-dependent amplifier of plaque progression and disruption, and clinical phenotyping as the bridge that determines when each pathway should be measured and treated. The sections are therefore sequenced to build a single argument from mechanism to implementation rather than restating the same concepts in parallel language.

## 2 Search strategy, study selection, and review scope

We conducted a structured narrative review rather than a formal systematic review. To improve transparency and reduce selection bias, the major thematic domains, key questions, and eligibility principles were defined for this review and then applied consistently during manuscript revision; however, no prospectively registered protocol was used. PubMed was searched from January 1, 2000 through March 1, 2025, and the electronic search was supplemented by backward citation screening together with targeted review of major cardiovascular-journal, guideline, and society webpages to capture pivotal outcome trials, consensus statements, and practice-changing evidence. Search strings iteratively combined disease-, mechanism-, biomarker-, and therapy-related terms, including “coronary atherosclerosis”, “inflammation”, “NLRP3”, “IL-1β”, “IL-6”, “NETs”, “apoB”, “remnant cholesterol”, “small dense LDL”, “lipoprotein(a)”, “PCSK9 inhibitor”, “ezetimibe”, “inclisiran”, “bempedoic acid”, “colchicine”, “canakinumab”, and “plaque vulnerability”. The review was restricted to English-language full-text publications. Two authors independently screened titles and abstracts, assessed potentially eligible full texts, and resolved disagreements by discussion and consensus. Priority was given to randomized cardiovascular outcomes trials, meta-analyses, guideline or consensus documents, and mechanistic studies with direct relevance to coronary plaque biology, thrombosis, biomarker-guided stratification, or therapeutic implementation. We excluded duplicate publications, conference abstracts lacking adequate methodological detail, purely descriptive reports without clear mechanistic or clinical relevance, and non-coronary studies unless they provided indispensable translational insight. Because this was a structured narrative review, PRISMA flow reporting and formal study-level risk-of-bias scoring were not applied; this limitation should be considered when interpreting evidence selection. Instead, prespecified eligibility principles, dual-author screening, and focused cross-checking of references were used to improve reproducibility, balance, and transparency. The final revised manuscript cites 147 references.

## 3 Overview of coronary atherosclerosis pathobiology

Atherosclerosis begins with endothelial dysfunction and increased permeability in regions of disturbed flow. ApoB-containing lipoproteins enter and are retained in the intima via proteoglycan binding, after which oxidative and enzymatic modification promotes innate immune sensing and leukocyte recruitment. Monocytes differentiate into macrophages, ingest modified particles through scavenger receptors, and become foam cells. As lesions mature, vascular smooth muscle cells (VSMCs) migrate and proliferate, producing extracellular matrix that forms the fibrous cap. Progressive necrotic core expansion—driven by cell death, defective efferocytosis, and ongoing lipid deposition—predisposes plaques to rupture or erosion, triggering thrombosis and clinical events.

### 3.1 Endothelial activation and leukocyte recruitment

Endothelial cells integrate hemodynamic stress, metabolic injury, and inflammatory stimuli. Upregulation of adhesion molecules (VCAM-1, ICAM-1, selectins) and chemokines promotes rolling, firm adhesion, and transmigration of leukocytes. This “gateway” step links risk factors (hyperlipidemia, smoking, diabetes) to immune-cell trafficking and lesion initiation.

### 3.2 Foam cells, immunometabolism, and necrotic core biology

Macrophage uptake of modified lipoproteins is coupled to inflammatory activation via metabolic stress, mitochondrial dysfunction, reactive oxygen species, and cholesterol crystal formation. Defective efferocytosis prevents clearance of apoptotic cells and amplifies inflammation, enlarging necrotic cores and weakening caps. These processes represent central mechanistic links between chronic inflammation and plaque vulnerability.

### 3.3 Plaque disruption and immunothrombosis

Rupture-prone plaques often feature large necrotic cores and thin fibrous caps with macrophage infiltration and proteolysis. Erosion may involve endothelial denudation and heightened immunothrombosis. Neutrophil extracellular traps (NETs), platelet activation, and coagulation converge to produce thrombosis and acute coronary syndromes, connecting inflammatory effector programs to clinical events.

## 4 Inflammatory drivers and pathways

**Figure 3:**
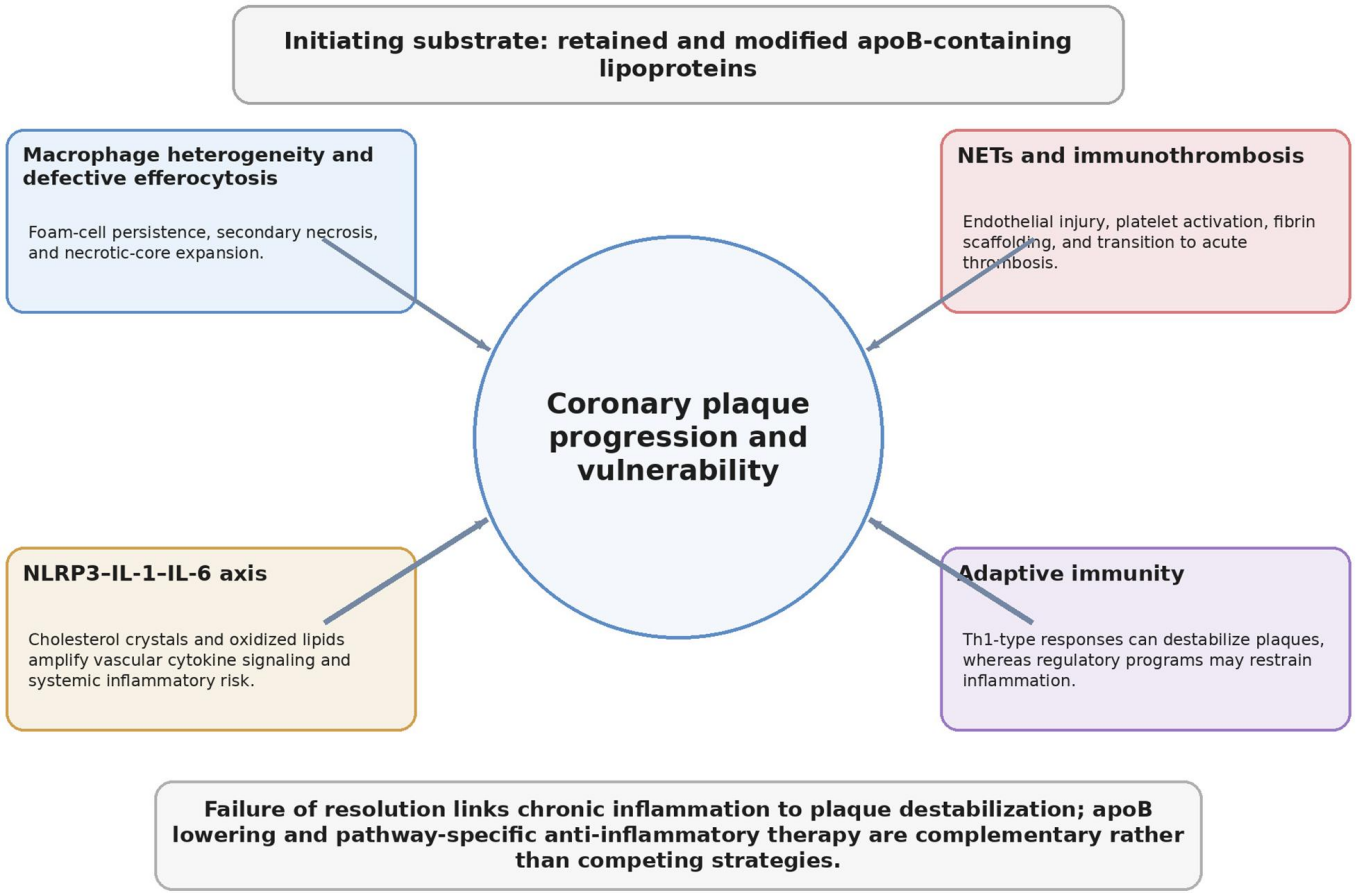
Key inflammatory pathways linking apoB retention to plaque vulnerability. Major modules include macrophage heterogeneity/efferocytosis failure, NET-driven immunothrombosis, NLRP3–IL-1–IL-6 signaling, and adaptive immunity.

Atherosclerosis is sustained by persistent stimuli (retained apoB lipoproteins) and failure of resolution (4–6, 15–23). Key inflammatory pathways linked to plaque progression and instability are summarized in Table 2, and a detailed mechanistic schematic is provided in Figure 3. Inflammation is expressed as endothelial activation, myeloid recruitment and activation, maladaptive immunometabolism, and prothrombotic programs coupling plaque inflammation to clinical events.

**Table 2:** Inflammatory pathways linked to plaque progression and instability.

Pathway	Key nodes	Plaque biology	Clinical translation
NLRP3–IL-1β	Crystals/oxlipids → NLRP3 → IL-1β	Amplifies inflammation; destabilization	CANTOS; colchicine
IL-6 axis	IL-6 signaling	Systemic inflammation; acute-phase response	Ziltivekimab; ZEUS ongoing
NETs	NETosis; platelet–leukocyte crosstalk	Endothelial injury; thrombosis	Associations; candidate targets
Efferocytosis failure	MerTK; “don't-eat-me” cues	Necrotic core expansion	Emerging targets
Failed resolution	Low SPMs; impaired circuits	Persistent inflammation	Pro-resolving strategies

### 4.1 Macrophage heterogeneity and efferocytosis failure

Single-cell and functional studies reveal multiple macrophage states, including lipid-handling programs, interferon-driven programs, and reparative programs (18, 19, 22–28). This heterogeneity matters because different states may drive foam-cell persistence, cytokine production, or resolution failure. Defective efferocytosis is a pivotal amplifier: impaired apoptotic-cell clearance promotes secondary necrosis, necrotic-core expansion, and ongoing inflammatory signaling. In addition to canonical receptor-level checkpoints, macrophage efferocytosis also appears to be shaped by transcriptional and epigenetic programs; notably, recent preprint evidence suggests that RBPJ-dependent chromatin regulation may modulate macrophage efferocytic capacity, linking cell-state programming directly to inflammation resolution (29). Because this line of evidence is still emerging, it should be interpreted as hypothesis-generating until broader peer-reviewed validation is available.

### 4.2 Neutrophils, NETs, and thromboinflammation

NETs are emerging contributors to plaque inflammation, endothelial injury, and thrombosis (30–33). Mechanistically, NET-associated DNA, histones, and proteases can disrupt endothelial integrity, amplify leukocyte recruitment, activate platelets, and provide a scaffold for fibrin-rich thrombus formation. Beyond generic thromboinflammation, accumulating human and experimental data now link NET-rich plaque microenvironments to features of plaque instability, supporting the concept that NETs participate in the transition from inflamed plaque to acute atherothrombotic events (30, 31, 33, 34).

### 4.3 NLRP3 inflammasome and IL-1–IL-6 axis

Cholesterol crystals and oxidized lipids activate NLRP3 inflammasomes, promoting IL-1β maturation and amplifying vascular inflammation (11, 12, 14, 23, 35–43). IL-1 signaling drives downstream IL-6 activity and acute-phase responses (CRP), providing a measurable axis of residual inflammatory risk. CANTOS provided proof-of-concept for IL-1β inhibition, while colchicine demonstrated outcome benefits in post-MI and chronic CAD, emphasizing pathway relevance.

### 4.4 Failed resolution and specialized pro-resolving mediators

Atherosclerosis can be conceptualized as non-resolving inflammation: persistent stimuli and defective efferocytosis prevent a return to homeostasis (18, 19, 22–27). Specialized pro-resolving mediators (SPMs) actively terminate inflammation and promote repair, offering a conceptual alternative to broad immune suppression.

Resolution-focused translation requires measurable readouts (18, 19, 22, 23, 25–27). Unlike LDL-C, resolution lacks a single scalable clinical marker. Candidate approaches include lipid mediator panels, efferocytosis-related signatures, and composite indices integrating systemic inflammation with plaque morphology. The therapeutic promise is substantial—enhancing resolution may stabilize plaques without the infection signal seen with some cytokine inhibitors—but clinical development will require careful dose-finding, safety monitoring, and validation of intermediate endpoints.

Non-resolving inflammation reflects more than defective efferocytosis (18, 19, 22, 23, 25–27). Resolution is an active process coordinated by specialized pro-resolving mediators (SPMs), stromal cell signals, and macrophage phenotypic switching. In plaques, persistent stimuli maintain inflammatory macrophage programs and constrain pro-resolving circuits, favoring necrotic core expansion and cap thinning. VSMCs and fibroblast-like stromal cells also participate by remodeling extracellular matrix, contributing to cap stability, and responding to cytokine and lipid mediator cues.

### 4.5 Endothelial mechanobiology and cardiometabolic stress

Metabolic stress magnifies this spatial vulnerability (1–3, 43). Hyperglycemia increases formation of advanced glycation end products and oxidative stress; smoking increases reactive species and endothelial dysfunction; and hypertension increases mechanical strain. Together, these exposures lower the threshold for endothelial activation and amplify the probability that retained particles will become modified and immunogenic. Clinically, this reinforces why comprehensive risk factor control remains essential even when lipid targets are achieved: reducing mechanical and metabolic stress reduces endothelial permissiveness and inflammatory responsiveness.

Atherosclerotic lesions cluster at arterial branch points and curvatures where disturbed flow creates a unique endothelial state (4–6, 15–17, 20, 21). Low and oscillatory shear stress induces endothelial transcriptional programs that favor permeability, oxidative stress, and leukocyte adhesion. These regions become “hot spots” for apoB particle entry and retention, converting systemic exposure into local inflammatory activation.

### 4.6 Adaptive immunity: antigen specificity, tolerance, and immune regulation

A key translational concept is antigen specificity (4–6, 15–17, 20, 21). Modified lipoproteins (e.g., oxidized LDL) and apoB-derived peptides can be recognized as antigens, creating an immunological bridge between lipid biology and adaptive responses. This raises the possibility of antigen-specific interventions—vaccination, immune tolerance induction, or epitope-focused antibody strategies—that modulate pathogenic immunity without broadly suppressing host defense. However, clinical translation remains early because dominant antigens in human coronary disease are incompletely defined, immune responses vary across disease stages, and durable immune modulation must be achieved without increasing infection or malignancy risks.

Adaptive immunity shapes plaque inflammation and stability through multiple, sometimes opposing, programs (1–3, 43). Th1-polarized responses and interferon-*γ* signaling amplify macrophage activation, promote expression of matrix-degrading enzymes, and can weaken fibrous-cap integrity. By contrast, regulatory T cells constrain inflammation via IL-10 and TGF-*β* and may promote more stable plaque phenotypes. B-cell subsets can be protective or pathogenic depending on the balance between natural antibodies that neutralize oxidized lipid epitopes and pro-inflammatory cytokine programs.

### 4.7 Immunothrombosis: platelets, complement, and coagulation as inflammatory effectors

Complement provides an additional link between inflammation and thrombosis (3, 43–49). Complement fragments can enhance leukocyte recruitment, modify endothelial permeability, and interact with tissue factor and coagulation cascades. Although complement activation is detectable in human plaques, it remains challenging to distinguish causal amplification from secondary activation during tissue injury. Future studies integrating proteomic signatures, lesion imaging, and clinical phenotyping may identify complement-dominant subgroups in whom targeted modulation could be beneficial.

While apoB retention initiates plaque inflammation, acute coronary syndromes occur when inflammation is converted into thrombosis (4–6, 15–17, 20, 21, 50–53). Immunothrombosis highlights this conversion: neutrophils, platelets, endothelial cells, and coagulation pathways form a feed-forward network. NETs can promote platelet activation, provide a scaffold for fibrin deposition, and expose histones and proteases that injure endothelium and propagate thrombin generation. Platelets are not merely hemostatic; they deliver chemokines, facilitate leukocyte recruitment, and stabilize platelet–leukocyte aggregates at sites of plaque disruption.

### 4.8 Pattern-recognition signaling and innate immune checkpoints beyond NLRP3

From a translational standpoint, broad receptor blockade is challenging because these pathways are essential for host defense and tissue homeostasis (4–6, 15–17, 20, 21). Nevertheless, understanding receptor-level priming has practical implications. First, it supports early, intensive apoB lowering: reducing the number of particles entering the wall reduces the probability of generating immunogenic ligands that prime innate pathways. Second, it suggests that anti-inflammatory therapies may be more effective when the priming stimulus is simultaneously reduced—helping explain why combining potent lipid lowering with pathway-relevant inflammation targeting is biologically plausible. Third, it motivates a search for selective checkpoints that can dampen pathological signaling without global immunosuppression, including metabolic regulators of macrophage activation and lesion-specific delivery platforms that restrict exposure to immune cells within plaques.

These receptor systems also explain why qualitative lipoprotein modification is so important (1–3, 43). Aggregated LDL and oxidized phospholipids are not merely ‘more cholesterol’; they are immunogenic ligands that shift macrophage programming toward pro-inflammatory, retention-permissive states. Hyperglycemia and advanced glycation end products can further enhance receptor signaling, especially in diabetes, and can impair cholesterol efflux by altering transporter expression and by remodeling HDL function. The end result is a self-reinforcing loop: modified particles intensify innate sensing; innate activation increases oxidative stress and further particle modification; and continued retention maintains a chronic danger-signal bath within the intima.

Inflammasome activation is a central, clinically relevant amplifier, but coronary inflammation is initiated and sustained by broader pattern-recognition signaling (4–6, 12, 15–17, 20, 21, 23, 36, 37, 40–43). Oxidized lipids and modified apoB particles engage scavenger receptors such as CD36 and SR-A, promoting foam-cell formation while simultaneously priming inflammatory transcriptional programs. Toll-like receptors (TLRs) and related innate receptors integrate these danger signals with metabolic stress, converging on NF-*κ*B activation, chemokine production, and recruitment of additional leukocytes. In this context, NLRP3 activation is often best viewed as a second-stage amplifier that requires priming: receptor-level signals induce pro–IL-1*β* expression, and crystalline or mitochondrial danger signals then trigger inflammasome assembly and cytokine maturation.

### 4.9 Inflammation reshapes lipid metabolism and lipoprotein function: bidirectional amplification

Mechanistically, bidirectional coupling supports combination strategies (4–6, 15–17, 20, 21, 24, 44–46). Reducing apoB exposure decreases the initiating stimulus, while targeted anti-inflammatory therapy may reduce hepatic acute-phase responses and improve lipoprotein handling indirectly. This also implies that monitoring should not be limited to a single axis: lipid targets can be achieved while inflammation remains high, and vice versa, motivating the dual-axis approach proposed in this review.

This bidirectionality has clinical implications (4–6, 15–17, 20, 21, 24, 44, 46, 54, 55). Patients with chronic inflammatory states—obesity-related inflammation, CKD, autoimmune disease, and chronic infections—may exhibit lipid phenotypes that are more atherogenic than LDL-C alone suggests, including higher apoB particle number, higher remnant burden, and impaired HDL function. These patterns can help explain why some patients experience recurrent events despite intensive LDL-C lowering: the inflammatory milieu can sustain particle production and promote qualitative modification and retention-prone behavior of apoB particles.

The lipid and inflammation hypotheses are often presented as parallel explanations, but biology indicates a bidirectional loop (4–6, 15–17, 20, 21, 24, 44–46, 54, 55). Retained apoB particles initiate inflammation; in turn, systemic and vascular inflammation remodels lipoprotein metabolism and function. Inflammatory cytokines can increase hepatic VLDL production, impair lipoprotein lipase activity, and alter clearance of triglyceride-rich particles, thereby increasing the pool of apoB-containing remnants capable of arterial entry. Inflammation also increases oxidative modification of lipoproteins and shifts HDL toward dysfunctional states, potentially reducing cholesterol efflux capacity and anti-inflammatory signaling.

### 4.10 VSMC plasticity, calcification, and fibrous-cap stability

From a translational perspective, VSMC biology reinforces the importance of combined strategies that reduce inflammatory protease activity while preserving or restoring fibrous-cap integrity (4–6, 15–23, 25–27). Intensive apoB lowering reduces the initiating stimulus; inflammation targeting may reduce matrix-degrading signaling; and resolution/efferocytosis enhancement may limit necrotic core expansion that destabilizes caps. Future imaging and biomarker approaches that capture cap biology and microcalcification—rather than focusing only on luminal stenosis—could improve precision selection of stabilization-focused therapies.

Calcification further complicates stability (49, 56–66). Microcalcifications within thin caps can increase local mechanical stress and promote rupture, whereas larger, consolidated calcification may in some contexts reflect more stable disease. Inflammation influences calcification by modulating VSMC osteogenic differentiation and by altering the balance between calcification inhibitors and promoters. Lp(a) biology is relevant here because Lp(a) and its oxidized phospholipid cargo have been linked to calcific processes in vascular and valvular disease, suggesting that inherited lipid risk may influence not only plaque growth but also tissue-level remodeling.

Vascular smooth muscle cells (VSMCs) are structural stewards of plaque stability, but they are also dynamic immune-metabolic participants (1–3, 43). Under inflammatory stress and lipid exposure, VSMCs can undergo phenotypic switching toward macrophage-like or osteogenic programs, altering extracellular matrix production and potentially weakening fibrous caps. This switching matters because a stable cap depends on adequate collagen synthesis and controlled protease activity; inflammatory cytokines and oxidative stress can shift VSMCs away from matrix maintenance and toward maladaptive states.

## 5 Lipid-related determinants beyond LDL-C

LDL-C lowering remains foundational, yet apoB particle burden, remnants, sdLDL, and Lp(a) refine atherogenic exposure and help explain residual risk (4–6, 15–17, 20, 21, 24, 44, 46, 50–60, 63–65). ApoB reflects particle number and can reveal discordance when LDL-C underestimates particle burden, common in insulin resistance. Remnant cholesterol reflects TRL remnant cholesterol content and is supported by genetic evidence for causality. Lp(a) identifies genetically mediated risk and is a rapidly evolving therapeutic frontier.

Until outcomes trials for Lp(a)-lowering are complete (4–6, 15–17, 20, 21, 56–60, 63–65), the actionable implication is risk amplification: intensify management of modifiable risks and reduce apoB exposure aggressively in patients with elevated Lp(a).

Measurement requires nuance: mg/dL and nmol/L assays are not interchangeable, and apo(a) isoform size influences results (4, 5, 16, 17). A practical approach is at least one lifetime measurement, with earlier testing in premature CAD or strong family history.

Lp(a) is genetically determined and can amplify risk through apoB-mediated retention, oxidized phospholipid cargo, and potentially prothrombotic apo(a) effects (4–6, 15–17, 20, 21, 50–53, 56–60, 63–65). Thus, Lp(a) integrates atherogenic, inflammatory, and thrombotic biology rather than acting as “LDL-C by another name.”.

### 5.1 LDL-C, non–HDL-C, and apoB: aligning clinical targets with causal exposure

Monitoring should match the dominant phenotype (4–6, 15–17, 20, 21, 24, 44–46, 50–60, 63–65, 67, 68). In particle-dominant phenotypes (high apoB/non–HDL-C), repeat lipid panels ensure sustained exposure reduction and adherence. In TRL-dominant phenotypes, triglycerides and remnant-C guide metabolic optimization and therapy selection. In genetically amplified phenotypes [high Lp(a)], monitoring focuses on achieving lower apoB exposure and controlling other risk factors while awaiting targeted Lp(a) therapy with proven outcomes. This structured monitoring approach prevents overreliance on a single marker and improves the plausibility that therapy is modifying the causal substrate of disease.

For routine care, “treat-to-exposure” is a useful framing: aim to minimize time spent above harmful atherogenic particle exposure levels (4–6, 15–17, 20, 21, 24, 44–46, 67, 68). In very-high-risk patients, this supports early combination therapy to achieve robust apoB exposure reduction rather than prolonged stepwise escalation. When apoB is unavailable, non–HDL-C can serve as a pragmatic surrogate of atherogenic exposure, particularly in hypertriglyceridemia.

### 5.2 Apob discordance phenotypes: insulin resistance, diabetes, and chronic kidney disease

In practice, LDL-C is sufficient for many patients when triglycerides are not elevated (4–6, 15–17, 20, 21, 24, 44–46, 67, 68). However, in metabolic dyslipidemia, LDL particles can be cholesterol-depleted such that LDL-C underestimates particle burden. In these discordant phenotypes, apoB (or non–HDL-C when apoB is unavailable) provides a biologically aligned view of exposure and can better explain persistent plaque progression despite apparently well-controlled LDL-C.

LDL-C is a validated therapeutic target, but it reflects cholesterol content rather than particle number (4–6, 15–17, 20, 21, 24, 44, 46, 54–60, 63–65, 67, 68). Because arterial retention is a particle-based process—each apoB-containing particle can enter the intima and become retained—metrics that better approximate particle burden can refine residual cholesterol risk. Non–HDL-C captures total atherogenic cholesterol (LDL plus remnants), and apoB directly approximates the number of atherogenic particles [LDL, remnants, IDL, and Lp(a)].

### 5.3 Remnant cholesterol and triglyceride-rich lipoproteins: particle biology and inflammatory coupling

A pragmatic strategy is to measure apoB when triglycerides are persistently elevated, when metabolic syndrome or diabetes is present, or when events occur despite low LDL-C (4–6, 15–17, 20, 21, 24, 44–46, 54, 55). If apoB remains high, intensification of particle-lowering therapy with combination regimens and control of TRL burden is mechanistically coherent.

ApoB discordance is common in insulin resistance, type 2 diabetes, and chronic kidney disease, where TRL flux and altered lipoprotein remodeling can increase particle number without proportionally increasing LDL-C (4–6, 15–17, 20, 21, 24, 44, 46, 54, 55). This produces a frequent clinical scenario: LDL-C meets target, but apoB remains elevated and event risk remains high.

### 5.4 Qualitative LDL atherogenicity: oxidation, glycation, aggregation, and sdLDL

A central translational point is that triglyceride concentration and apoB-containing particle burden are related but not interchangeable (24, 44–46, 54, 55, 69–72). A therapy may lower plasma triglycerides largely by changing lipid content within particles or by altering circulating kinetics without materially reducing the number of apoB-containing remnants available for arterial retention. By contrast, strategies that reduce remnant/TRL apoB burden are more likely to attenuate arterial entry, endothelial activation, and downstream inflammatory coupling. This distinction helps explain why triglyceride-lowering trials have been heterogeneous and why pathway-specific TRL programs must be interpreted through the lens of apoB particle biology rather than triglyceride concentration alone.

Remnant cholesterol represents cholesterol carried by triglyceride-rich lipoproteins and remnants (4–6, 15–17, 20, 21, 24, 44–46, 54, 55). Remnant particles are apoB-containing and can be retained in the arterial wall. In addition, TRL lipolysis products can activate endothelium and myeloid cells, providing a plausible inflammatory coupling that amplifies plaque progression in insulin resistance phenotypes.

### 5.5 HDL function: why raising HDL-C failed and what remains clinically relevant

From a translational standpoint, sdLDL is rarely a primary therapeutic target (4, 5, 16, 17); instead, it signals the need to address upstream drivers of metabolic dyslipidemia and particle burden while maintaining intensive LDL-C lowering.

Beyond quantity, qualitative modification of apoB particles influences inflammatory potential (4–6, 15–17, 20, 21, 24, 44, 46, 54, 55). Oxidation and aggregation increase uptake by scavenger receptors and intensify innate immune sensing; glycation in diabetes can further enhance atherogenicity. Small dense LDL is more oxidation-prone and often coexists with remnant elevation, serving as a marker of broader atherogenic remodeling.

### 5.6 Lipoprotein(a): measurement nuance, biology beyond LDL, and therapeutic thresholds

While HDL function assays are not standardized for routine care (4, 5, 16, 17), the concept remains clinically useful: high inflammatory burden may be accompanied by dysfunctional HDL despite normal HDL-C, reinforcing the need for integrated lipid–inflammation strategies.

HDL-C concentration correlates inversely with risk, yet pharmacologic HDL-C raising has not consistently reduced events, suggesting that HDL function matters more than HDL quantity (4, 5, 16, 17). Cholesterol efflux capacity, antioxidant activity, and anti-inflammatory signaling represent key functional features that can be impaired by systemic inflammation.

### 5.7 Translating Lp(a) biology to therapy: modality trade-offs and implementation questions

This framing also clarifies why some cardiometabolic interventions show broad cardiovascular benefit: they act on multiple nodes simultaneously—hepatic lipid flux, endothelial function, inflammatory tone, and thrombosis propensity (4–6, 15–17, 20, 21, 24, 44–46, 54, 55). For a practical review, the key message is integration: intensive apoB reduction remains foundational, but it should be embedded within a cardiometabolic strategy that reduces TRL flux and systemic inflammatory priming. This combined approach is especially important in diabetes, obesity, CKD, and MASLD/NAFLD, where residual cholesterol and inflammatory risks often coexist and reinforce each other.

Weight loss and improved glycemic control therefore have mechanistic relevance beyond their traditional risk-factor roles (4–6, 15–17, 20, 21, 24, 44, 46, 54, 55). Reducing visceral adiposity decreases pro-inflammatory cytokine production and may improve HDL function and insulin sensitivity, indirectly lowering remnant particle burden. Improved glycemic control reduces glycation-related lipoprotein modification and may lessen endothelial activation. In chronic kidney disease, reducing uremic inflammatory drivers and optimizing volume and blood pressure can decrease inflammatory stress, though lipid phenotypes can remain complex and require continued focus on apoB exposure.

Coronary risk is amplified when lipid exposure and inflammation are embedded within a broader cardiometabolic syndrome (4–6, 15–17, 20, 21, 24, 44–46, 54, 55). Insulin resistance increases hepatic VLDL production, raises TRL remnant burden, and promotes LDL-C–apoB discordance. At the same time, adipose tissue inflammation increases systemic cytokine tone and acute-phase responses, and it contributes to endothelial dysfunction through oxidative stress and impaired nitric oxide bioavailability. Thus, cardiometabolic control can be interpreted as ‘upstream substrate reduction’ (fewer apoB-containing remnants and fewer retention events) plus ‘upstream response reduction’ (lower inflammatory priming).

### 5.8 Practical targets, monitoring, and ‘treat-to-exposure’ philosophy

Key threshold questions remain (4–6, 15–17, 20, 21, 56–60, 63–65). First, how much Lp(a) lowering is required to reduce events, and does this threshold depend on baseline Lp(a) concentration and overall absolute risk? Second, what is the time-to-benefit for Lp(a) reduction, and how should it be integrated with aggressive apoB lowering that has immediate plaque-stabilizing effects? Third, will event reduction be mediated primarily through atherogenesis slowing, plaque stabilization, or thrombotic modulation? These questions matter because they determine which patients should be prioritized [e.g., those with very high Lp(a) and premature events] and how therapy should be sequenced relative to standard LDL/apoB-lowering strategies.

The rapid expansion of Lp(a)-lowering modalities creates new implementation questions (56–60, 63–65). RNA-based approaches (antisense and siRNA) can achieve large reductions with durable suppression, potentially enabling infrequent dosing paradigms. Oral small-molecule approaches that interfere with particle assembly could broaden accessibility and adherence. These modalities differ in dosing frequency, tolerability, cost, and potential off-target effects; therefore, outcomes trial results must be interpreted not only for efficacy but also for feasibility in real-world secondary prevention.

### 5.9 Metabolic therapies and the lipid–inflammation axis: why cardiometabolic control stabilizes plaques

Finally, timing interacts with biology (4–6, 15–23, 56–60, 63–65). Reducing apoB exposure early may prevent priming of innate immune programs and reduce the probability that plaques evolve toward non-resolving inflammatory states. In later disease, apoB reduction still stabilizes plaques, but adjunct strategies that target inflammation, immunothrombosis, or resolution failure may be required for maximal event reduction. This time-dependent view supports a life-course approach to prevention that integrates lipid exposure, inflammatory burden, and risk modifiers such as Lp(a).

Cumulative exposure also reframes residual risk in secondary prevention (4, 5, 16, 17). Many patients have already accumulated decades of exposure before their first event, and even very low LDL-C cannot instantly erase established plaque. Thus, early intensive therapy in high-risk individuals and strong persistence over time are essential. For clinicians, the practical takeaway is to treat ‘early and durably’: avoid prolonged periods above exposure targets, consider combination therapy when gaps are large, and address adherence as a primary therapeutic task rather than an afterthought.

A unifying explanation for the consistent benefit of LDL/apoB lowering across trials is cumulative exposure (4–6, 15–17, 20, 21). Atherosclerosis develops over decades; therefore, the area under the curve of atherogenic particle exposure—how high, and for how long—drives lifetime risk. This concept clarifies why earlier initiation and sustained adherence produce larger absolute benefits than late intensification, even when the achieved LDL-C is similar at a single time point.

## 6 Clinical translation: biomarkers, imaging, and residual-risk stratification

Clinicians increasingly need a practical bridge between mechanistic biology and bedside decision-making. Residual-risk assessment should therefore ask two separate but interacting questions: is the dominant unresolved problem continued exposure to atherogenic apoB particles, persistent inflammatory activation, or both? LDL-C, non–HDL-C, apoB, remnant-related measures, and Lp(a) address the exposure side of the problem, whereas hsCRP and selected cytokine-pathway markers help phenotype inflammatory activation; imaging is most useful when lesion-level uncertainty remains after blood-based phenotyping.

Monitoring should be pragmatic: repeat lipids to ensure sustained exposure reduction (16, 17, 43, 46, 47), reassess inflammatory biomarkers when an anti-inflammatory strategy is contemplated or initiated, and use imaging selectively when it will change management.

A phenotype-guided workflow helps reduce conceptual redundancy in both the manuscript and clinical practice. Rather than repeatedly invoking a broad framework, the useful clinical sequence is to verify adherence and secondary causes, intensify apoB lowering first, document whether meaningful inflammatory activity persists after stabilization, and then consider targeted anti-inflammatory therapy only when residual inflammatory biology remains demonstrable and clinically relevant.

### 6.1 Residual inflammatory risk biomarkers: hsCRP, IL-6 axis, and pragmatic interpretation

For inflammation targeting, reassess hsCRP after stabilization and after initiation of an anti-inflammatory strategy to confirm biological response (16, 17, 43, 46, 47). Because hsCRP is non-specific, repeated measures and clinical evaluation are crucial: a lack of hsCRP reduction may reflect nonadherence, inadequate dosing, or an alternative inflammatory driver not addressed by the selected therapy. Imaging should be reserved for situations where it will change management—recurrent events, major biomarker discordance, or evaluation of high-risk plaque features in selected contexts.

Response assessment should verify that therapies are modifying the intended biology (4–6, 15–17, 20, 21, 24, 44–46, 54, 55, 67, 68). For lipid therapy, recheck lipids after initiation or intensification to confirm sustained LDL-C/non–HDL-C reduction and, when available, apoB reduction. For TRL-focused interventions, recheck triglycerides and consider remnant-C; interpret changes in the context of weight and glycemic control.

### 6.2 Lipid phenotyping beyond LDL-C: when to measure apoB, remnant-C, and Lp(a)

More proximal biomarkers may improve specificity (3, 35, 38, 39, 43). IL-6 pathway activity is linked to outcomes and is a direct therapeutic target in ongoing trials. Composite biomarkers and cell-based indices may complement hsCRP in selected contexts, particularly when interpreting immunothrombotic biology.

hsCRP is the most accessible clinical biomarker for systemic inflammation and has been used for trial enrichment (16, 17, 43, 46, 47, 73). Persistently elevated hsCRP after stabilization and exclusion of secondary causes can identify patients with residual inflammatory risk and potentially larger absolute benefit from pathway-relevant anti-inflammatory therapy. However, hsCRP is not pathway-specific, so repeated measurement and clinical context are essential to avoid misclassification. In practice, biomarker selection should follow the clinical question rather than a generic label: hsCRP for scalable screening and serial follow-up, IL-6-pathway markers when cytokine-axis intervention or trial enrollment is being considered, apoB/non–HDL-C when discordant particle exposure is suspected, and selective imaging when lesion phenotype may change management. This purpose-driven approach improves patient stratification by distinguishing burden-dominant, inflammation-dominant, and mixed phenotypes.

### 6.3 Imaging for plaque burden and vulnerability: how to use CTA, IVUS/OCT, and molecular imaging

Lp(a) should be measured at least once in adulthood to identify inherited risk (56–60, 63–65). This information guides lifetime prevention intensity, supports earlier combination therapy, and helps interpret residual risk when LDL-C appears controlled.

A practical lipid phenotyping approach identifies scenarios where LDL-C alone may underestimate causal exposure (4–6, 15–17, 20, 21, 24, 44–46, 54, 55, 67, 68). ApoB is most informative when triglycerides are elevated, metabolic syndrome or diabetes is present, or events occur despite low LDL-C. Non–HDL-C offers a widely available surrogate of total atherogenic cholesterol, and remnant-C can highlight TRL-related exposure when triglycerides remain elevated.

### 6.4 Integrating biomarkers and imaging into patient stratification and monitoring

Molecular imaging approaches remain largely research tools due to cost and standardization barriers (16, 17, 43, 46, 47), but they are conceptually important for validating inflammatory targets and bridging mechanisms to clinical phenotypes.

Imaging complements blood-based markers by visualizing plaque burden and composition (3, 18, 19, 22, 23, 43–49, 74–76). Coronary CTA quantifies plaque burden and can identify high-risk plaque features. IVUS and OCT provide higher-resolution characterization of plaque architecture and cap thickness in selected patients undergoing invasive evaluation.

### 6.5 Clinical phenotypes and discordance: how biomarkers and imaging change decisions

In clinical practice, imaging is most useful in discordant or high-uncertainty scenarios rather than as a routine escalation test (4–6, 15–17, 20, 21, 35, 38, 39, 43, 67, 68). When clinical risk remains high despite apparently controlled lipids, imaging may reveal high plaque burden or vulnerability features that justify more intensive therapy. When anti-inflammatory treatment is being considered, imaging can help determine whether active plaque vulnerability still appears biologically plausible despite adequate substrate reduction. Used this way, biomarkers help answer which pathway is active, whereas imaging helps show how that biology is being expressed within the artery. In research, combining imaging with biomarker phenotyping can reduce uncertainty and provide a clearer bridge from mechanism to individualized prevention.

However, imaging endpoints must be interpreted cautiously (18, 19, 22, 23, 46–49, 74–76). Not all imaging changes translate into clinical benefit, and different modalities capture different biological layers. CTA is well suited for total burden and noninvasive plaque phenotyping but has limited resolution for cap thickness. OCT provides exquisite cap resolution but is invasive and typically limited to selected segments. IVUS provides plaque volume measures but less detailed cap architecture. Molecular imaging can, in principle, capture inflammatory activity, but standardization, cost, and uncertain incremental value remain barriers. Thus, imaging should be chosen based on the biological hypothesis and the clinical question, rather than used as a generic ‘more data’ approach.

A recurring challenge in coronary prevention is that clinical events are relatively infrequent on modern background therapy, especially in shorter trials, making outcomes studies expensive and slow (16, 17, 43, 46, 47). Imaging can therefore serve as a valuable intermediate endpoint that helps validate whether a therapy is plausibly modifying plaque biology in the desired direction. For lipid lowering, reductions in atheroma volume and favorable shifts in plaque composition provide mechanistic reassurance consistent with exposure-time biology. For inflammation targeting, lesion-level imaging of high-risk plaque features, inflammatory activity proxies, or cap-stabilization markers can help confirm that systemic biomarker changes translate into local plaque effects.

### 6.6 Practical monitoring and response assessment: what to re-check and when

Over the longer term, multi-omic profiling and spatially resolved biology may identify dominant plaque programs—NET-driven immunothrombosis, macrophage inflammasome activation, VSMC cap failure, or resolution deficits—that can be matched to targeted therapies (16–19, 22, 23, 37–50, 67, 68). However, translation will require standardization, external validation, cost containment, and proof that phenotype-guided therapy improves outcomes beyond guideline-based care. Until then, the practical goal is to use existing tools in a disciplined way: measure what changes management, verify biological response to therapy, and avoid overinterpreting non-specific markers in isolation.

A realistic path to precision phenotyping is incremental rather than disruptive (35, 38, 39, 43, 56–60, 63–65). In the near term, dual-axis phenotyping (particle burden and inflammatory burden) provides a mechanistically coherent and feasible approach. The next step is adding pathway specificity where it is most likely to change decisions: IL-6 pathway markers when IL-6–axis inhibition is considered; Lp(a) and oxidized phospholipid-related markers when inherited risk dominates; and selective lesion imaging in recurrent events or suspected high-risk plaque.

Current clinical biomarkers are powerful because they are scalable, but they are imperfect proxies of lesion biology (4–6, 15–17, 20, 21). LDL-C and apoB reflect circulating exposure, not arterial retention rates; hsCRP reflects systemic inflammation, not plaque-specific pathway activity; and imaging often captures structure rather than dynamic inflammatory signaling. These limitations can lead to discordant signals—patients with low LDL-C but active plaque inflammation, or patients with high hsCRP due to non-atherosclerotic causes.

### 6.7 Translational thresholds and workflow integration

A practical residual-risk framework must specify thresholds that are clinically actionable and feasible across care settings (11, 14, 43). For residual cholesterol risk, LDL-C remains the primary target, but particle-aware metrics such as apoB or non–HDL-C are especially informative when LDL-C is discordant in insulin-resistant phenotypes; in such cases, intensification should be guided by the magnitude of particle excess and the patient's absolute risk (16, 17, 20, 21, 77). For residual inflammatory risk, persistence of hsCRP elevation after stabilization and exclusion of non-atherosclerotic drivers provides a pragmatic entry point, but escalation should still depend on whether the signal is reproducible, clinically meaningful, and linked to a plausible therapeutic pathway. This threshold-based framing strengthens translational relevance by connecting biomarkers to concrete treatment decisions rather than to descriptive risk labeling alone.

Workflow integration can be operationalized as a stepwise algorithm: confirm adherence and exclude secondary drivers; reduce apoB exposure with early combination therapy when the gap to target is large; phenotype residual risk using apoB/non–HDL-C, remnant-C, and one-time Lp(a) measurement; then reassess inflammatory burden and consider pathway-relevant anti-inflammatory therapy in patients with persistent inflammation and high absolute risk (16, 17, 44, 46, 56, 57, 67, 68, 77). As summarized in Figure 2, this sequencing aligns measurement with mechanism, sharpens patient stratification, and avoids escalating therapies that do not address the dominant biology.

Implementation thresholds should be explicit for emerging therapies (46–49, 67–74). For IL-6–axis inhibition and Lp(a)-lowering agents, trial programs are increasingly enriched for high-risk phenotypes; in practice, adoption will likely prioritize patients with recurrent events despite controlled LDL-C/apoB and confirmed residual inflammatory or inherited risk signals, with careful safety and access considerations (23, 35, 38, 39, 41–43, 51, 63–65). Embedding these thresholds into electronic order sets and follow-up loops—repeat lipids for exposure, repeat hsCRP for inflammatory response, and selective imaging when it changes management—translates trial-level efficacy into real-world effectiveness.

## 7 Therapeutic perspectives: lowering lipids and targeting inflammation

**Figure 4:**
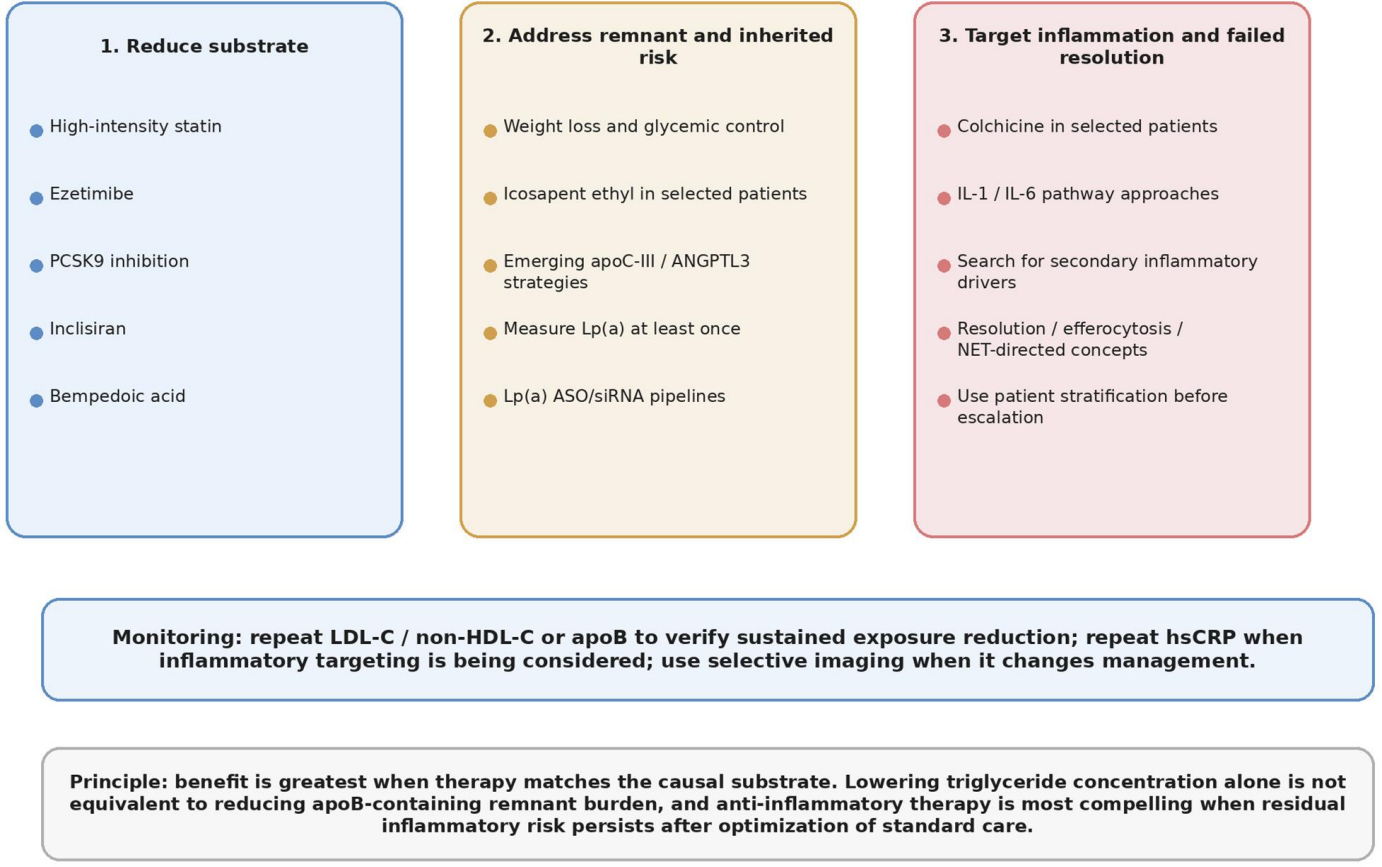
Therapeutic roadmap across the lipid–inflammation continuum. Major therapeutic domains include apoB lowering, remnant/Lp(a)-directed strategies, and pathway-relevant inflammation or resolution-focused interventions.

Outcome trials support both lipid lowering and inflammation targeting (4–6, 15–17, 20, 21, 24, 35, 38, 39, 43, 44, 46, 54–60, 63–65). Therapeutic strategies addressing residual cholesterol and inflammatory risks are summarized in Table 3, landmark outcome trials are summarized in Table 4, and Figure 4 provides a consolidated therapeutic roadmap. To reduce redundancy, the discussion below focuses on treatment selection, modality-specific trade-offs, and implementation rather than restating the mechanistic framework developed in earlier sections.

**Table 3:** Therapies addressing residual cholesterol and inflammatory risks.

Strategy	Example agents	Outcome evidence	Notes
ApoB/LDL lowering	Statins; ezetimibe; PCSK9i; inclisiran; bempedoic acid	Reduced MACE (multiple RCTs)	Backbone; intensify to targets
TRL/remnant risk	Icosapent ethyl; apoC-III/ANGPTL3 inhibitors (emerging)	REDUCE-IT; outcomes pending	PROMINENT null for pemafibrate
Anti-inflammatory	Colchicine; canakinumab; IL-6 axis (emerging)	COLCOT/LoDoCo2; CANTOS; ZEUS ongoing	Monitor safety; interactions
Lp(a)-targeted (emerging)	ASO/siRNA; muvalaplin	Large Lp(a) reductions in phase 2	Outcomes awaited

**Table 4:** Selected landmark outcome trials supporting lipid- and inflammation-targeted strategies.

Trial	Population	Intervention	Key finding
IMPROVE-IT	Post-ACS	Ezetimibe+statin	Reduced CV events
FOURIER	ASCVD	Evolocumab+statin	Reduced MACE
ODYSSEY OUTCOMES	Post-ACS	Alirocumab+statin	Reduced MACE
CLEAR Outcomes	Statin-intolerant	Bempedoic acid	Reduced MACE
CANTOS	Prior MI + hsCRP elevated	Canakinumab	Reduced events; infection risk
COLCOT/LoDoCo2	Post-MI/chronic CAD	Low-dose colchicine	Reduced events
REDUCE-IT	High risk + TG elevated	Icosapent ethyl	Reduced ischemic events
PROMINENT	Diabetes + TG elevated	Pemafibrate	No event reduction

Precision prevention will likely integrate genetics [including Lp(a) and polygenic risk], biomarker-defined residual risk axes, and imaging-defined plaque phenotypes (4–6, 15–17, 20, 21, 50–53, 56–60, 63–65, 67, 68). Near-term opportunities are pragmatic: use apoB/non–HDL-C, Lp(a), hsCRP, and selective imaging to guide intensity and combination therapy while awaiting definitive outcomes trials for emerging therapies.

Beyond IL-1/IL-6, multiple upstream targets are under investigation, including inflammasome components, chemokine axes, and immune–metabolic regulators (18, 19, 22, 23, 25–27, 36–43). Resolution enhancement—via SPM pathways or restoration of efferocytosis—offers an attractive route to stabilize plaques without broad immunosuppression, but translation will depend on scalable biomarkers and safety data.

### 7.1 A pragmatic stepwise algorithm for dual-axis residual risk management

Step 1: Confirm adherence, exclude secondary causes, and optimize lifestyle and comorbidities (weight, diabetes, hypertension, CKD management, smoking cessation) (4–6, 8–10, 15–17, 20, 21, 24, 44–46, 54–60, 63–65, 67, 68, 78–80). Step 2: Intensify apoB lowering to reach stringent secondary-prevention targets using combination therapy (statin+ezetimibe, then PCSK9 inhibition; bempedoic acid or inclisiran as indicated). Step 3: Phenotype residual cholesterol risk with apoB/non–HDL-C and remnant-C when triglycerides are elevated; measure Lp(a) once to identify inherited amplification.

Step 4: After stabilization, measure hsCRP to phenotype residual inflammatory risk; repeat to confirm persistence and evaluate secondary drivers (4–6, 11, 14–17, 20, 21, 35, 38, 39, 43, 73). Step 5: For patients with persistent inflammatory risk and high absolute risk despite controlled apoB exposure, consider pathway-relevant anti-inflammatory therapy with explicit attention to infection risk, tolerability, and interactions. Step 6: Reassess response and persistence of control—lipids for substrate, hsCRP for inflammation—using imaging selectively when it will change management. This algorithm operationalizes the mechanistic framework into a feasible clinical workflow.

### 7.2 Lipid-lowering therapy as substrate removal: sequencing, adherence, and exposure-time biology

Because retention events accumulate over time, earlier and sustained reduction in apoB exposure yields greater lifetime benefit (4–6, 15–17, 20, 21). This supports avoiding prolonged periods above target in very-high-risk patients—favor earlier combination therapy when baseline risk is high, LDL-C is far from goal, or apoB discordance suggests under-recognized particle exposure.

ApoB exposure reduction is the most established lever for preventing coronary events (4–6, 8–10, 15–17, 20, 21, 78–80). High-intensity statins remain foundational; when targets are not met, combination therapy with ezetimibe and then PCSK9 inhibition provides robust additional apoB reduction and outcome benefit. Bempedoic acid should be explicitly included in this framework because CLEAR Outcomes demonstrated cardiovascular benefit in statin-intolerant patients, making it an important oral option for LDL-C and apoB lowering when statins are not tolerated or when additional reduction is needed (10). ACLY biology may also intersect with macrophage lipid synthesis and plaque remodeling: myeloid Acly deficiency promotes a more stable plaque phenotype in experimental atherosclerosis, and additional emerging preclinical evidence—including recent preprint work in myocardial infarction models—suggests that activated macrophage fatty-acid synthesis can propagate pathogenic fibroblast expansion after injury (10, 81, 82). These mechanistic observations remain hypothesis-generating and should not be interpreted as evidence that bempedoic acid is currently an established anti-inflammatory plaque therapy independent of its lipid-lowering effect. Inclisiran's twice-yearly dosing also remains attractive for adherence.

### 7.3 Targeting TRL/remnant risk: aligning interventions with particle reduction and biology

Emerging TRL therapies (apoC-III and ANGPTL3 inhibition) are promising because they can reduce TRL particle burden substantially (4–6, 15–17, 20, 21, 24, 44, 46, 54, 55). For these approaches, outcomes trials should confirm meaningful reductions in apoB-containing remnant exposure and test mechanistically aligned intermediate endpoints.

Management of TRL/remnant risk should align interventions with causal exposure (24, 44–46, 54, 55). Lifestyle and metabolic optimization remain first-line, particularly weight loss and glycemic control. Among pharmacologic options, the goal is not triglyceride lowering *per se*, but reduction of apoB-containing remnant burden and its downstream inflammatory consequences. Icosapent ethyl has outcomes evidence in selected high-risk patients, whereas several triglyceride-focused interventions have been neutral in contemporary settings, underscoring that lowering triglyceride concentration without materially reducing atherogenic particle number is often insufficient.

### 7.4 Targeting inflammation: what worked, what failed, and how to select patients

The IL-6 axis is a leading candidate for upstream targeting of residual inflammatory risk (4–6, 15–17, 20, 21, 35, 38, 39, 43). In the near term, inflammatory targeting is most defensible in patients with persistently elevated hsCRP after stabilization and risk factor optimization, particularly when recurrent events occur despite well-controlled apoB exposure.

Anti-inflammatory therapy should not be discussed as a uniform class effect (11–14, 42, 43, 83, 84). CANTOS demonstrated that selective IL-1β inhibition can reduce recurrent events in post-MI patients with hsCRP ≥2 mg/L, but the benefit came with clinically important infection and sepsis concerns, so net benefit is most plausible only in carefully selected patients with high absolute risk and clearly documented residual inflammation. Low-dose colchicine is easier to deploy and reduced events in post-MI and chronic coronary disease trials, yet routine use is constrained by gastrointestinal intolerance, renal/hepatic considerations, discontinuation burden, toxicity risk in vulnerable patients, and clinically relevant interactions with strong CYP3A4 or P-glycoprotein inhibitors. Negative or attenuated trials are equally informative: CIRT did not suppress IL-1β, IL-6, or hsCRP and did not improve outcomes, underscoring that cardiovascular benefit requires engagement of a causal inflammatory pathway rather than nonspecific immune modulation. The practical implication is that anti-inflammatory therapy should be phenotype-guided, biology-confirmed, and safety-filtered rather than used as a blanket add-on to contemporary secondary prevention.

### 7.5 Combination strategies: the dual-axis matrix in real-world secondary prevention

Implementation should prioritize safety and adherence. Emerging therapeutic pipelines relevant to residual risks are summarized in Table 5, and these programs emphasize modality-specific trade-offs and implementation thresholds (7–12, 14, 43, 44, 46). For lipid therapy, ensure tolerability and long-term persistence; for inflammation targeting, exclude secondary inflammatory conditions, monitor adverse effects, and reassess biomarker response to confirm pathway modulation.

**Table 5:** Emerging therapeutic pipelines relevant to residual cholesterol and inflammatory risks.

Target	Modality	Examples	Stage	Notes
Lp(a) synthesis	ASO/siRNA	Pelacarsen; olpasiran; lepodisiran	Phase 2–3	Large reductions; outcomes ongoing/awaited
Lp(a) formation	Oral small molecule	Muvalaplin	Phase 2	Oral dosing; large reductions
IL-6 axis	Monoclonal antibody	Ziltivekimab	Outcomes (ZEUS)	CKD+hsCRP enrichment; monthly dosing
TRL metabolism	ASO/antibody	ApoC-III; ANGPTL3 inhibitors	Phase 2–3	Outcomes evolving
Resolution/efferocytosis	Multiple	SPM analogs; efferocytosis enhancers	Early	Aim to restore resolution

Patients with high residual cholesterol risk and high residual inflammatory risk are candidates for maximal apoB lowering plus pathway-relevant anti-inflammatory therapy, because absolute benefit potential is greatest when both axes remain uncontrolled (4–6, 15–17, 20, 21). Patients with high cholesterol but low inflammation may benefit most from intensifying apoB reduction, whereas those with low apoB but persistent inflammation require evaluation for modifiable drivers and consideration of anti-inflammatory strategies.

### 7.6 Interpreting trial heterogeneity and modality-specific trade-offs

Similarly, triglyceride-focused trial discordance emphasizes the need to align interventions with causal exposure (4–6, 15–17, 20, 21, 24, 44–46, 56–60, 63–65). Some strategies lower triglyceride concentration without meaningfully reducing apoB-containing particle number or without modifying inflammatory coupling, leading to neutral outcomes. In contrast, interventions that reduce remnant apoB burden more substantially—or exert additional favorable effects on plaque biology—are more biologically plausible candidates for event reduction in selected cohorts. For Lp(a), early-phase data show large reductions, but event reduction will depend on duration and magnitude of lowering, baseline risk, and whether therapy modifies thrombosis and plaque biology in addition to atherogenesis. A translational review should therefore encourage readers to interpret trials through a unified lens: does the intervention measurably reduce the causal substrate (apoB particles and their atherogenic subclasses) and/or does it measurably dampen atherothrombotic inflammatory programs with acceptable safety?

The divergence between successful and unsuccessful anti-inflammatory trials can be interpreted through a translational framework (11–14, 23, 35–43, 83, 84). At least five variables appear to determine clinical success: enrichment for residual inflammation at baseline, engagement of a causal pathway, timing relative to ACS vs. stable disease, intensity of background lipid lowering, and tolerability sufficient to preserve long-term adherence. CANTOS succeeded in a biomarker-enriched population and achieved downstream cytokine suppression; CIRT enrolled a less inflamed population and left IL-1β/IL-6/hsCRP unchanged; colchicine trials differed in phenotype, timing, discontinuation burden, and competing medication interactions. Taken together, the trial record suggests that outcomes diverge not because inflammation is unimportant, but because pathway selection, patient phenotype, background therapy, and safety-related persistence determine whether anti-inflammatory biology can be translated into clinical benefit. These contrasts explain why positive anti-inflammatory trials cannot simply be generalized across all coronary artery disease populations, and why future studies should explicitly pair pathway selection with biomarker confirmation and patient phenotype.

The modern trial landscape illustrates a core principle: mechanistic plausibility is necessary but not sufficient—clinical benefit depends on population selection, background therapy, achieved biological effect, and safety (4–6, 15–17, 20, 21, 46–49). For lipid lowering, benefit is relatively predictable because apoB exposure is causal and measurable; the magnitude of event reduction scales with achieved exposure reduction and time. For inflammation targeting, heterogeneity is larger because ‘inflammation’ is not a single pathway; different interventions modulate distinct nodes with different trade-offs.

### 7.7 Safety, tolerability, and implementation constraints

Before prescribing colchicine, clinicians should review kidney function, hepatic impairment, frailty, baseline cytopenias, and interacting medications—especially strong CYP3A4 or P-glycoprotein inhibitors—because toxicity rises when clearance is impaired (11, 14, 43, 84). For cytokine-axis inhibition, infection history, vaccination status, and competing causes of CRP elevation should be evaluated before attributing residual risk to plaque biology alone. These safety filters explain why anti-inflammatory therapy is best positioned as a selective second-layer strategy after optimal substrate removal rather than as universal background therapy for all patients with coronary disease.

Real-world implementation requires attention to safety and feasibility (4–6, 8, 9, 15–17, 20, 21, 78–80). For LDL/apoB lowering, clinicians must address statin intolerance with structured rechallenge, alternative dosing, and evidence-based add-ons. PCSK9 inhibitors and inclisiran introduce access and adherence considerations; shared decision-making and systems-level support (prior authorization workflows, adherence reminders) often determine real-world success.

### 7.8 Follow-up, implementation, and conversion of efficacy into real-world benefit

Follow-up should verify biological response: repeat lipids to confirm sustained substrate reduction; reassess hsCRP when inflammatory targeting is contemplated or initiated; and escalate evaluation for persistent discordance (e.g., unexpected apoB elevation or persistent inflammation) by searching for secondary causes or considering selective imaging (4–6, 15–17, 20, 21, 67, 68). This disciplined follow-up approach converts trial-level efficacy into real-world effectiveness and supports rational sequencing of combination therapy.

When adding colchicine, counsel patients about gastrointestinal intolerance and review interacting medications, particularly strong CYP3A4 or P-gp inhibitors that may increase toxicity risk (11, 14, 43). In renal impairment, consider risk–benefit carefully and monitor more closely. If cytokine-axis inhibition becomes clinically available for atherosclerosis, baseline infection risk assessment, vaccination status review, and clear monitoring plans will be essential to keep safety trade-offs acceptable.

In secondary prevention, the greatest preventable loss of benefit often arises from gaps in implementation rather than gaps in evidence (4–6, 8, 9, 15–17, 20, 21, 67, 68, 78–80). A pragmatic prescribing approach starts with ensuring tolerance and persistence: confirm statin adherence, address side effects with structured rechallenge or alternative dosing, and use add-on therapies early when LDL-C/apoB gaps are large. For PCSK9 inhibitors or inclisiran, establish access pathways and adherence support because intermittent use undermines exposure-time benefit.

## 8 Discussion

This review supports a clinically useful synthesis: coronary risk is best understood through interacting but non-identical axes of apoB particle burden, inflammatory pathway activation, and failure of resolution. The key translational task is therefore not to restate these mechanisms, but to match them to patient selection, biomarker choice, and implementable treatment sequencing. Integration of genetics [Lp(a), polygenic risk], immune programming (trained immunity/CHIP), and imaging-defined plaque phenotype may further refine precision targeting, but only if workflows are cost-effective and improve outcomes beyond guideline-based care (16, 17, 50–53, 56–60, 63–65, 67, 68, 85–89). Pragmatic trials embedded in healthcare systems and implementation science will therefore be essential to translate dual-axis strategies into durable reductions in coronary events.

The next phase of coronary prevention research should prioritize bridging mechanistic insights to scalable implementation (4–6, 15–23, 25–27, 35, 38, 39, 43, 67, 68). First, trials should prespecify stratification around apoB/non–HDL-C, hsCRP/IL-6 pathway activity, Lp(a), and imaging-defined plaque phenotype so that the biology driving benefit can be identified rather than inferred *post hoc*. Second, biomarker selection should be pathway-matched: hsCRP for broad inflammatory enrichment, IL-6-axis markers when cytokine targeting is contemplated, apoB or remnant-related markers when particle exposure is central, and lesion imaging when local plaque phenotype is likely to alter treatment sequencing. Third, resolution biology requires standardized measurement—SPM panels, efferocytosis signatures, and lesion-level readouts—to move from concept to intervention.

### 8.1 Why triglyceride lowering does not always translate into event reduction

Future TRL trials should quantify apoB and remnant-C change (90–95), stratify by metabolic phenotype, and include intermediate endpoints consistent with reduced inflammatory stimulation. Clinically, this argues for evidence-based selection of therapies rather than assuming that all triglyceride-lowering strategies are equivalent.

Genetic and mechanistic evidence supports remnant/TRL causality, yet trials differ because interventions vary in whether they reduce apoB-containing particle exposure and whether they modulate inflammatory coupling. Triglyceride concentration is a surrogate; reductions do not necessarily imply fewer atherogenic particles or less arterial retention.

### 8.2 Immunothrombosis as the final common pathway to acute coronary syndromes

Translational priorities include standardization of immunothrombotic biomarkers and (96–101) validation of targets that reduce thrombosis without compromising host defense. In the near term, indirect modulation—via substrate removal and pathway-relevant inflammatory control—remains the most feasible route to reduce immunothrombotic propensity.

The conversion of plaque inflammation into thrombosis is the proximate cause of acute coronary syndromes. NETs, platelet activation, endothelial injury, and coagulation form a feed-forward network that couples inflammatory effector programs to thrombus formation.

### 8.3 Resolution biology—therapeutic promise and measurement challenges

A major gap is measurement: scalable clinical surrogates of resolution are lacking (61, 102–106). Developing validated resolution biomarkers and imaging endpoints will be essential for translating resolution-enhancing strategies and for determining whether they can stabilize plaques with fewer safety trade-offs than cytokine inhibition.

Resolution is an active program that terminates inflammation and promotes repair, mediated by specialized pro-resolving mediators and efficient efferocytosis. Resolution failure provides a mechanistically coherent explanation for necrotic core expansion and plaque vulnerability.

### 8.4 Genetics-informed prevention and the special case of lipoprotein(a)

Therapeutic pipelines—ASO/siRNA approaches and oral particle-formation inhibition—are (107–112) progressing quickly. Outcomes trials will define the required magnitude and duration of lowering and identify the highest-yield clinical populations for implementation.

Lp(a) identifies inherited residual risk and integrates atherogenic, inflammatory, and potentially prothrombotic biology. At least one lifetime Lp(a) measurement is practical and can trigger earlier and more intensive management of modifiable risks, including aggressive apoB exposure reduction.

### 8.5 Upstream hematopoiesis—trained immunity and clonal hematopoiesis (CHIP)

Translation will require scalable phenotyping and clear thresholds for intervention (113–117). If upstream inflammatory inhibition proves effective and safe, immune-programming–enriched phenotypes may become high-yield targets for precision prevention strategies.

Trained immunity and CHIP highlight that residual inflammatory risk can reflect durable immune programming, not just transient biomarker elevation. These mechanisms may help explain persistent inflammation in older patients and variability in response to standard lipid lowering.

### 8.6 Imaging endpoints as translational bridges

The key implementation question is when imaging changes management. A pragmatic model (66, 118–121) is biomarker-first risk phenotyping with selective imaging for discordant cases, recurrent events, or research settings evaluating new therapies.

Imaging quantifies plaque burden and vulnerability features, providing a bridge between mechanisms and clinical phenotypes. CTA enables noninvasive phenotyping; IVUS/OCT provide high-resolution characterization in selected patients undergoing invasive evaluation.

### 8.7 Implementation priorities for the next generation of trials and guidelines

Trial design should increasingly incorporate enrichment for residual-risk phenotypes (122–127), verification of on-target pathway modulation, and intermediate endpoints that map to mechanisms (plaque composition, inflammatory signatures, or resolution readouts). These steps will help translate mechanistic advances into durable reductions in coronary events on top of contemporary background care.

Across lipid and inflammation domains, the highest-yield near-term priority is phenotype-guided treatment pathways that are measurable, safe, and scalable. This includes harmonized use of apoB/non–HDL-C for particle burden, routine Lp(a) testing at least once, repeated hsCRP assessment when inflammatory targeting is considered, and selective imaging when it can change treatment intensity or sequencing.

### 8.8 Outstanding controversies and near-term priorities

This field is advancing rapidly (33–131), but several issues remain unsettled. First, the magnitude of benefit achievable by adding anti-inflammatory therapy on top of modern intensive lipid lowering—and the patient groups in whom benefit clearly outweighs risk—requires clearer definition. Second, triglyceride-lowering strategies continue to yield heterogeneous trial results, emphasizing the need to focus on particle burden and causal pathways rather than triglycerides alone. Third, for Lp(a) therapies, the magnitude and duration of lowering required for event reduction remain to be proven. Finally, scalable phenotyping tools for plaque inflammation and resolution are still evolving, limiting precision targeting in routine care.

### 8.9 Research agenda: bridging mechanism to implementation

To better bridge mechanism to implementation, future trials should routinely quantify both particle exposure and inflammatory activity, including apoB/non–HDL-C as substrate metrics and hsCRP with pathway-specific markers (e.g., IL-6 signaling) when relevant, so that biological on-target effects can be linked to plaque phenotypes and clinical outcomes (16, 20, 21, 38, 39, 77, 132–135).

In parallel, (18, 19, 35–50, 65, 67, 68) the field needs scalable readouts of resolution biology and immunothrombosis, and pragmatic health-system workflows that support adherence, rapid intensification for very-high-risk phenotypes, and equitable access to advanced therapies (136–139).

### 8.10 Take-home messages for clinicians and investigators

First, apoB-containing lipoproteins initiate coronary disease by arterial retention; therefore, exposure reduction remains the most reliable strategy for durable risk reduction (4–6, 15–17, 20, 21, 24, 35, 38, 39, 43, 44, 46, 54–60, 63–65, 67, 68). LDL-C is a useful target, but apoB and non–HDL-C can better reflect particle burden when discordance is likely, and remnant-C and Lp(a) refine residual cholesterol risk.

Second, residual inflammatory risk is real and modifiable, but pathway specificity and safety determine clinical usefulness (11–14, 35, 38–43, 73). hsCRP is a practical screening tool, while the IL-6 axis represents a leading upstream target under outcomes testing; however, anti-inflammatory therapy should be reserved for patients in whom the inflammatory signal is reproducible and the expected benefit outweighs infection, tolerability, and interaction-related risks.

Third, the lipid and inflammation hypotheses should be operationalized as complementary axes rather than competing explanations (3–6, 15–23, 43, 85–89). Patients high on both axes have the greatest absolute benefit potential from combined strategies, whereas discordant profiles call for more selective escalation. Fourth, implementation matters: scalable workflows—apoB/non–HDL-C for particle burden, lifetime Lp(a) testing, repeated hsCRP in selected patients, and selective imaging when it changes management—are available today and can improve clinical decisions even before next-generation therapies mature (140–142).

### 8.11 Cost, equity, and health-system implementation: making precision prevention scalable

Embedding these tiers into health-system workflows—standardized order sets, decision support based on structured residual-risk phenotyping, and follow-up reminders—can improve adherence and persistence, which are often the main determinants of long-term benefit (23, 35, 41, 43–46). Such implementation science is essential to translate rapidly expanding lipid–inflammation biology into durable reductions in coronary events across diverse patient populations.

A pragmatic approach is tiered implementation (4–6, 15–17, 20, 21, 56–60, 63–65, 67, 68). Tier 1 uses universally available tools: statins, ezetimibe, blood pressure and glycemic control, smoking cessation, and measurement of LDL-C/non–HDL-C and hsCRP in selected patients. Tier 2 adds apoB and Lp(a) testing where available to refine exposure and inherited risk (142). Tier 3 adds higher-cost therapies and selective imaging for the highest-risk phenotypes [recurrent events, high plaque burden, high Lp(a), persistent inflammatory risk] where absolute benefit is greatest (136, 140, 141).

Many of the most effective contemporary therapies—PCSK9 inhibition, long-acting RNA-based agents, and emerging Lp(a)-lowering modalities—raise cost and access challenges (8, 9, 56–60, 63–65, 78, 79). If precision prevention is implemented only in highly resourced settings, population-level event reduction will be limited. Therefore, a translational review should explicitly acknowledge that scalability and equity are part of the scientific problem: mechanisms only matter clinically when they can be acted upon (136, 140, 143).

## 9 Conclusion

Coronary atherosclerosis should be managed as a disease of retained apoB-containing particles, maladaptive inflammation, and their interaction within the plaque microenvironment. In practice, the most defensible sequence is to lower apoB exposure first and durably, then determine whether clinically meaningful inflammatory activity persists, and finally use selective imaging when lesion-level information is likely to change treatment intensity or sequencing. ApoB/non–HDL-C, remnant-related measures, one-time Lp(a) testing, and hsCRP each add value when they are matched to the biological question they are meant to answer.

Future progress will depend less on repeating the concept of residual risk and more on converting it into a workable clinical workflow. The next phase should prioritize explicit patient stratification, pathway-matched therapy, confirmation that the intended biology has actually been modified, and pragmatic delivery models that preserve safety, adherence, and access. Ongoing IL-6-pathway, TRL-targeting, and Lp(a)-lowering trials may refine this framework, but current practice should remain anchored in durable apoB reduction, selective biomarker-guided anti-inflammatory escalation, and implementation strategies that make effective therapy sustainable in routine care.
